# A case of multicentric gliomas in both supra- and infratentorial regions with different histology: a case report

**DOI:** 10.1186/s12957-016-0907-4

**Published:** 2016-05-26

**Authors:** Akihiro Inoue, Takanori Ohnishi, Shohei Kohno, Yosuke Mizuno, Riko Kitazawa, Yawara Nakamura, Shiro Ohue

**Affiliations:** Department of Neurosurgery, Ehime University School of Medicine, 454 Shitsukawa, Toon, Ehime 791-0295 Japan; Division of Diagnostic Pathology, Ehime University Hospital, 454 Shitsukawa, Toon, Ehime 791-0295 Japan

**Keywords:** Epidermal growth factor receptor, Multicentric gliomas, p53, Primary glioblastoma, Secondary glioblastoma

## Abstract

**Background:**

Multicentric gliomas are well-separated tumors in different locations of the brain, without anatomical continuity between lesions. We report a rare case of multicentric gliomas that occurred in both supra- and infratentorial regions with different histopathology.

**Case presentation:**

A 27-year-old man was admitted to our hospital with mild motor weakness of the right leg. Magnetic resonance imaging (MRI) showed a large tumor occupying the left insula, extending to the left basal ganglia, so tumor resection was performed. Histological diagnosis was diffuse astrocytoma. Tumor cells showed sporadic immunoreactivity for p53 and negative immunostaining for epidermal growth factor receptor (EGFR). Postoperative course was uneventful, and adjuvant therapy was not performed. At 7 months after surgery, MRI disclosed a left cerebellar tumor displaying an irregular ring formation on enhancement with gadolinium (Gd) and marked peritumoral edema. MRI studies including T2-weighted imaging demonstrated that this paravermian tumor had no contact with the initial left insular tumor. In addition, MRI studies of the whole neuraxis, cytological examination of the cerebrospinal fluid, and neurological findings demonstrated that no dissemination had occurred through the subarachnoid space or as intracerebral metastases. Therefore, the second surgery was performed. Histological diagnosis was glioblastoma. Immunohistochemistry revealed that most tumor cells were positively stained for both p53 and EGFR but negatively stained for isocitrate dehydrogenase 1 (IDH1).

**Conclusions:**

We reported a case of multicentric gliomas occurring in both supra- and infratentorial regions with different histopathology. Immunohistochemical examinations suggest that different genetic pathways may participate in the occurrence of these tumors.

## Background

Multicentric gliomas are well-recognized entities, but their occurrence is still rare. Among these, gliomas arising from both supra- and infratentorial regions with different histology are very rare [1–4]. We report the case of a patient with multicentric gliomas whose tumors occurred in both supra- and infratentorial regions with different histological types. One was diffuse astrocytoma in the left insula, and the other was glioblastoma in the cerebellum. We present the case and discuss the possible pathogeneses of multicentric gliomas.

## Case presentation

**Fig. 1:**
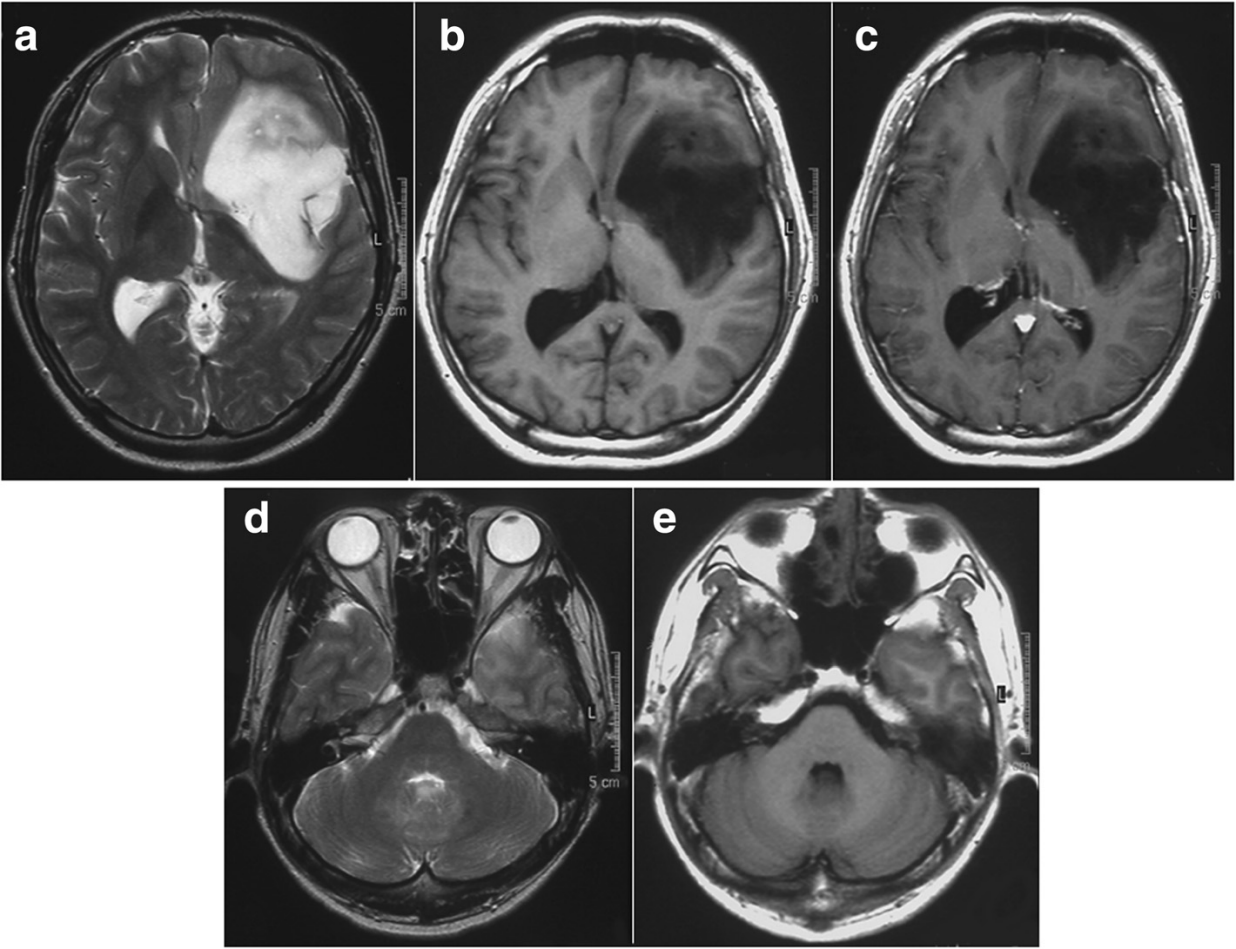
Preoperative axial T2-weighted (**a**), T1-weighted (**b**), and gadolinium (Gd) enhanced T1-weighted (**c**) magnetic resonance (MR) images showing a tumor mass in the left insula with a mild mass effect. The tumor was not enhanced on the administration of Gd. Axial T2-weighted (**d**) and Gd enhanced T1-weighted (**e**) MR images obtained at the same time showing no tumor in the cerebellum

**Fig. 2:**
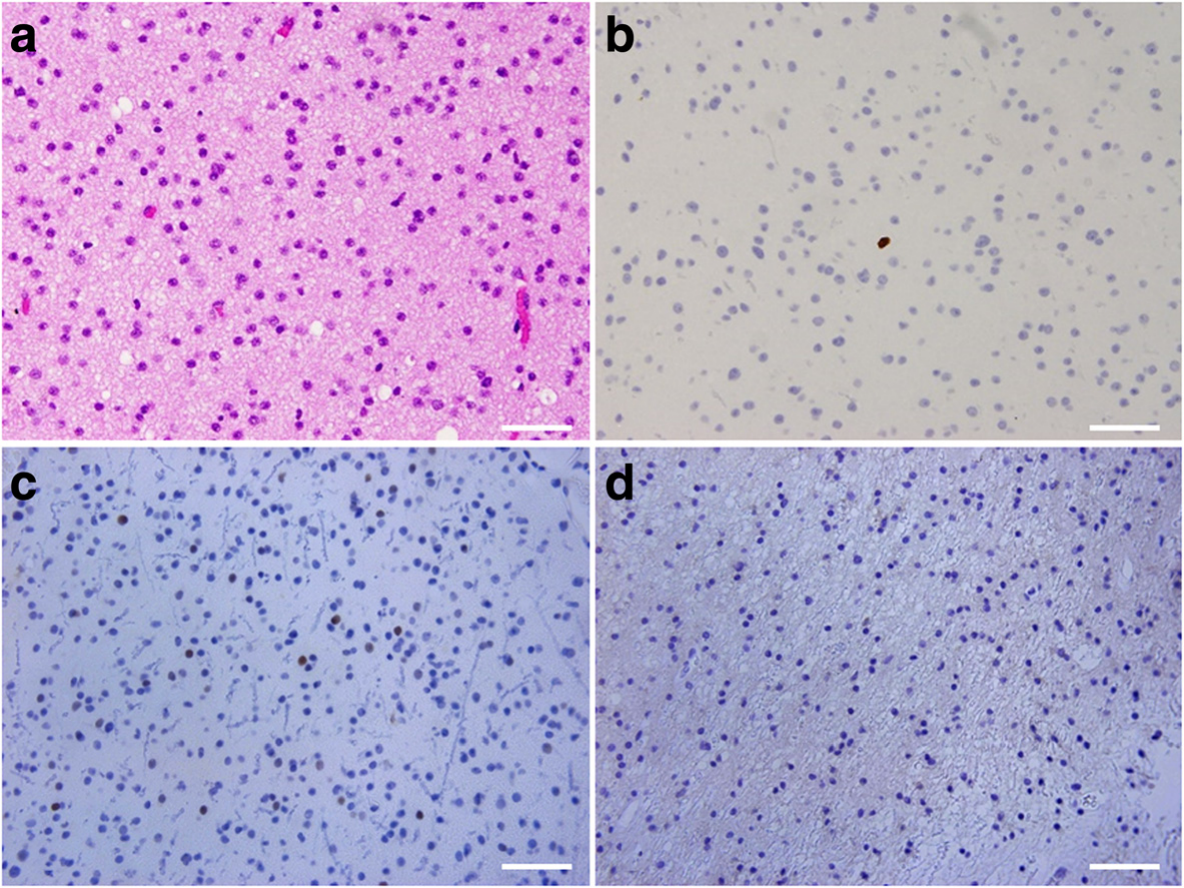
Photomicrographs showing histopathology of the left insular tumor. Tumor cells presenting nuclear atypia such as small and semicircular nuclei were increased in number with microcystic stroma. No mitotic cells were observed (hematoxylin and eosin ×400, *scale bar*: 50 μm) (**a**). Immunohistochemistry for MIB-1 showing a very low Ki-67 (MIB-1) proliferation-related labeling index (1 % or less) (**b**). Immunohistochemistry for p53 (**c**) and epidermal growth factor receptor (EGFR) (**d**) revealing sporadic immunoreactivity of tumor cells for p53, but no immunoreactivity for EGFR. **b**–**d** ×400, *scale bar*: 50 μm

A 27-year-old man began to experience disorientation and visited his family doctor. Although magnetic resonance imaging (MRI) showed an abnormal mass in the left insula, extending to the left basal ganglia, he was followed without therapy. The tumor gradually increased in size, and he was referred to our department. Neurological examination on admission revealed clear consciousness and no abnormality in cranial nerve functions other than mild weakness of the right leg (manual muscle test: 4/V). MRI on initial admission showed a tumor mass (diameter, 70 mm) extending from the left insula to the basal ganglia. The lesion was hypointense on T1-weighted imaging and hyperintense on T2-weighted imaging. The lesion was not enhanced on administration of gadolinium (Gd) (Fig. 1a–c). No other lesions were identified in the brain, including the cerebellum (Fig. 1d, e). Cerebral angiography disclosed no apparent tumor staining or abnormal vessels. As intraoperative histological examination revealed that the left insular tumor was a low-grade glioma, the tumor was partially resected under the electrophysiological monitoring using a motor-evoked potential to avoid serious neurological complications. Postoperative histological diagnosis was diffuse astrocytoma (World Health Organization (WHO) grade II astrocytoma) (Fig. 2a). Immunohistochemical studies showed that Ki-67 (MIB-1) proliferation-related labeling index was very low at 1 % or less, and tumor cells revealed sporadic immunoreactivity for p53, but a negative staining for epidermal growth factor receptor (EGFR) (Fig. 2b–d). Postoperative course was uneventful, and adjuvant therapy was not performed because of a very low MIB-1 labeling index in the tumor.

**Fig. 3:**
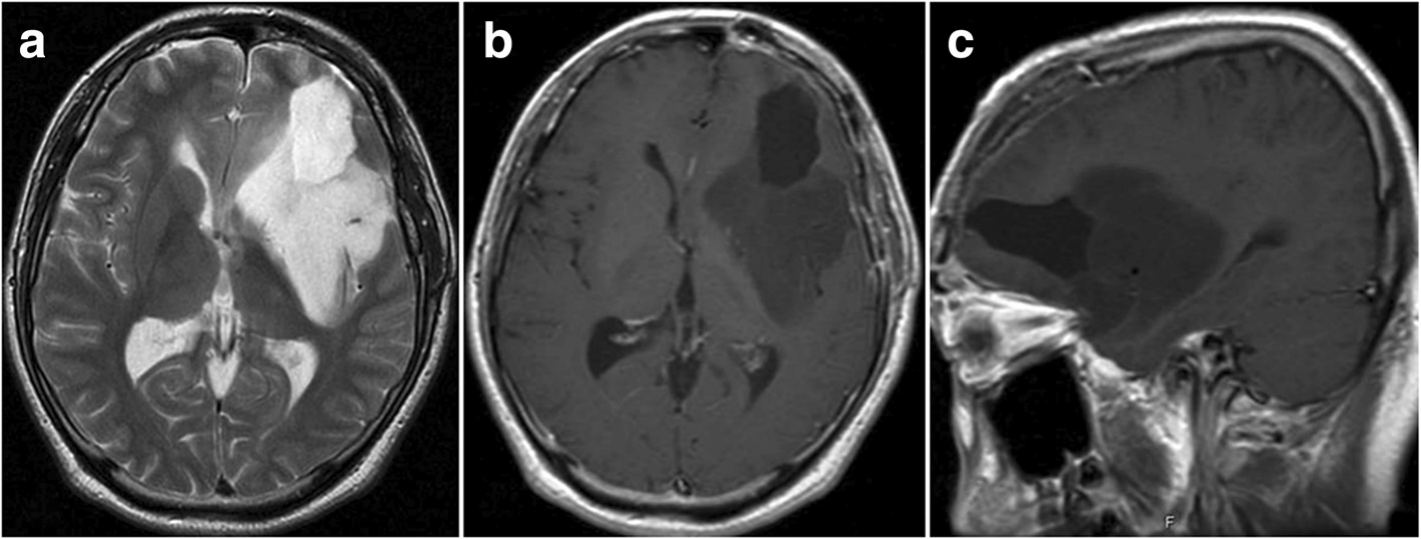
MR images at 3 months after the first surgery showing no growth of the left frontal tumor and no lesion at the other sites including the cerebellum. Axial T2-weighted image (**a**), axial (**b**), and sagittal (**c**) Gd-enhanced T1-weighted MR images

**Fig. 4:**
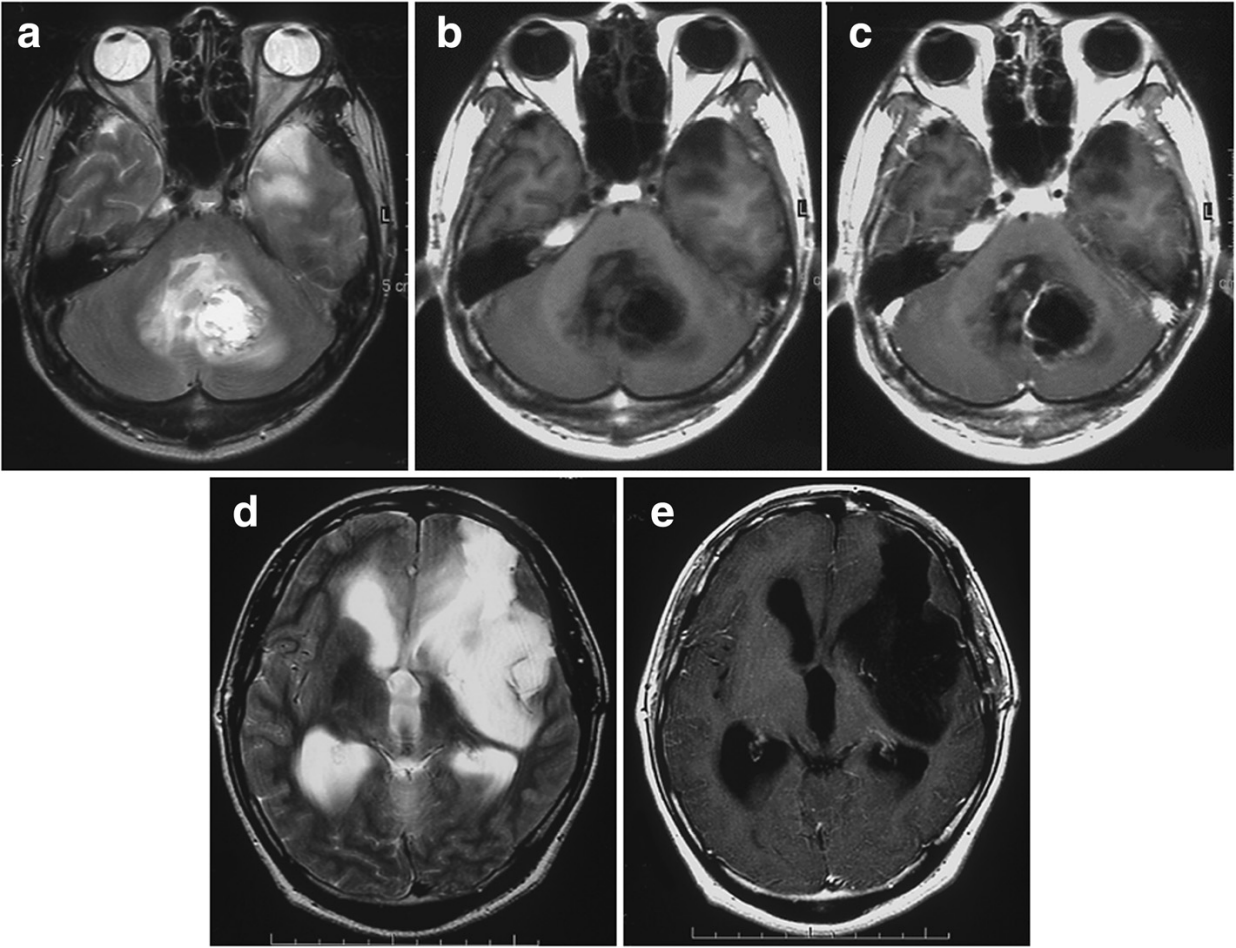
MR images at 7 months after the first surgery showing occurrence of a large tumor at the paravermian region of the left cerebellum. The tumor was enhanced in a ring-like fashion by Gd and accompanied by marked peritumoral brain edema. Axial T2-weighted (**a**), T1-weighted (**b**), and Gd-enhanced T1-weighted (**c**) MR images. MR images disclosing no tumor growth of the left insular tumor and hydrocephalus. Axial T2-weighted (**d**) and Gd-enhanced T1-weighted (**e**) MR images

**Fig. 5:**
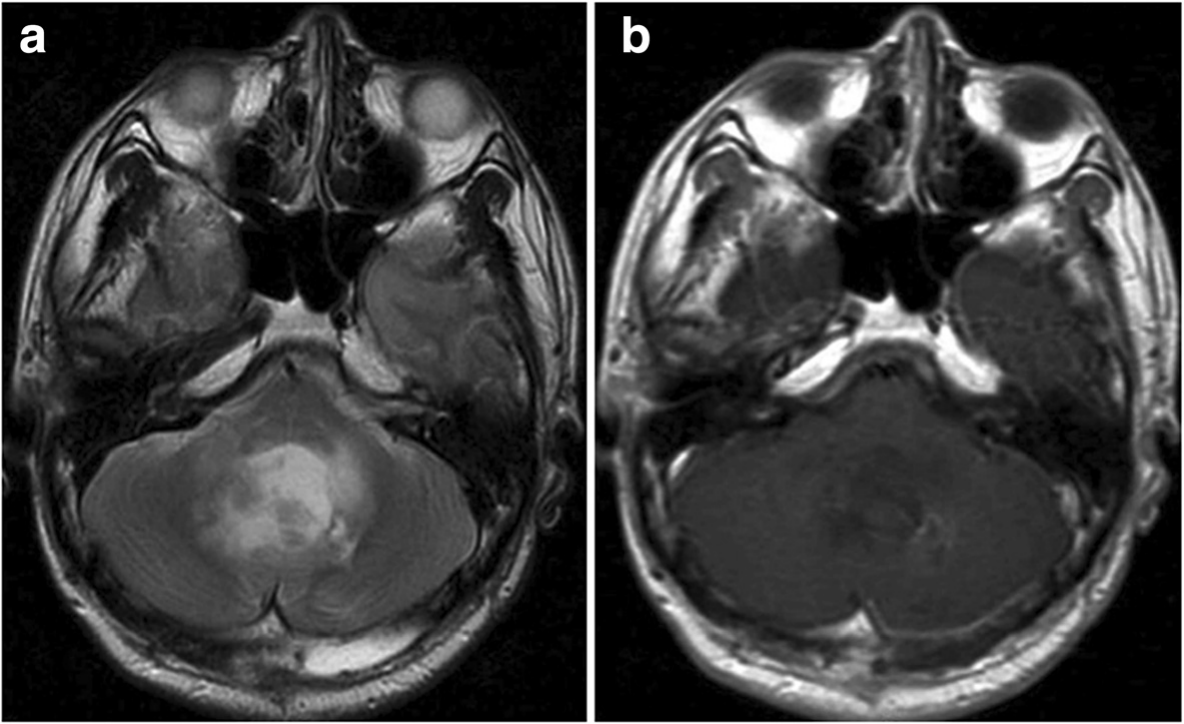
MR images at 1 week after the second surgery revealing total resection of the cerebellar tumor. Axial T2-weighted (**a**) and Gd-enhanced T1-weighted (**b**) MR images. Brain edema around the vermis still moderately persists, and the fourth ventricle remains obstructed

**Fig. 6:**
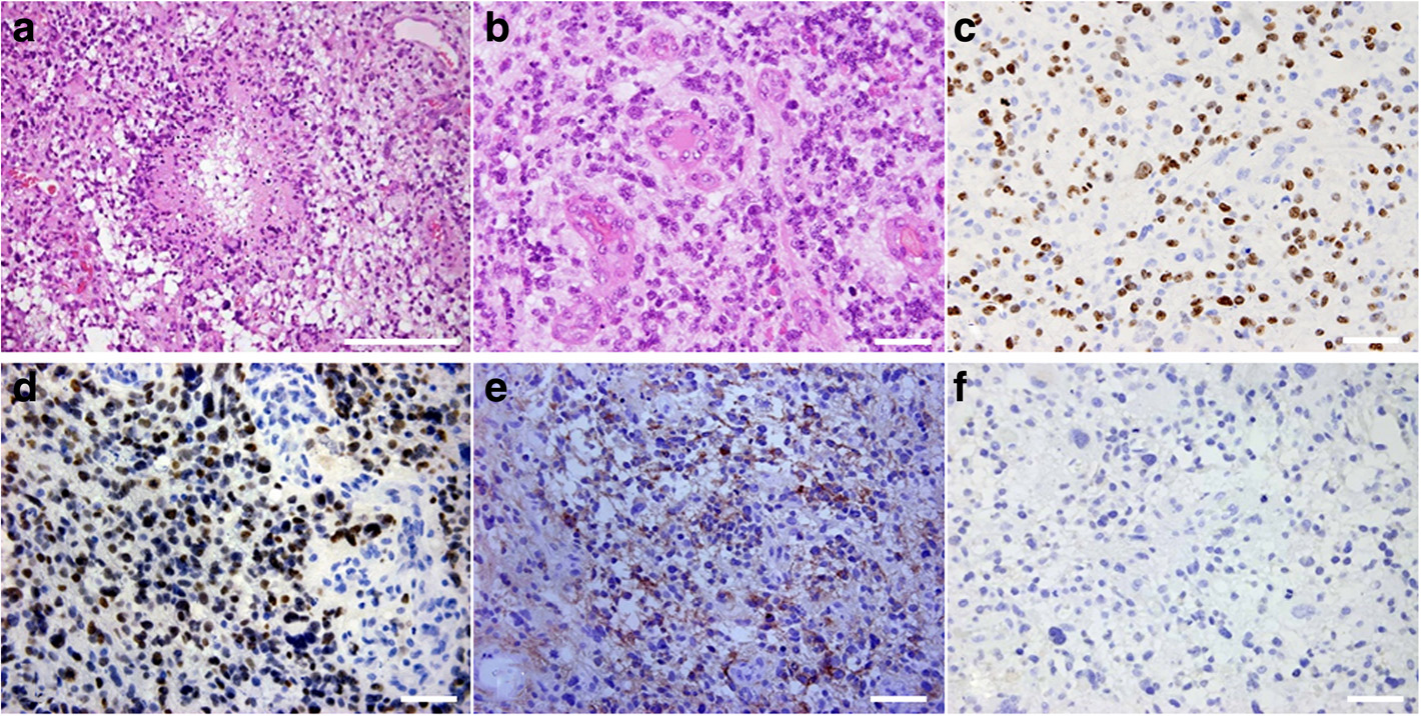
Photomicrographs showing histopathology of the cerebellar tumor. Microscopic histology (**a**) displayed the tumor with high cellularity, increased tumor vessels, and a pseudopalisading of tumor cells around large necrosis. The microscopic view with high magnification (**b**) revealed tumor cells with marked cellular and nuclear anaplasia and the prominent proliferation of vascular endothelial cells and necrosis (hematoxylin and eosin, **a** ×100, *scale bar*: 400 μm, **b** ×400, *scale bar*: 50 μm). Immunohistochemistry for MIB-1 showing a high Ki-67 (MIB-1) labeling index (42.8 %) (**c**). Immunohistochemistry for p53 (**d**) and EGFR (**e**) disclosing strongly positive immunoreactivity of tumor cells for both molecules, but a negative staining for IDH1 (**f**). **c**–**f** ×400, *scale bar*: 50 μm

MRI performed 3 months after surgery did not show any growth of the left insular tumor or any other lesion (Fig. 3). At 7 months after surgery, the patient presented with headache, nausea, and cerebellar symptoms. MRI disclosed a left cerebellar tumor displaying an irregular ring formation on enhancement with Gd and marked peritumoral edema (Fig. 4a–c). The residual supratentorial tumor remained unchanged compared to MRI 7 months earlier (Fig. 4d, e). Total resection of the cerebellar tumor was performed (Fig. 5) and a ventriculo-peritoneal shunt was placed because of persisting hydrocephalus. Histological examination revealed that the tumor showed high cellularity, cellular and nuclear anaplasia, and mitotic figures and also had prominent microvascular proliferation and large necrosis often accompanied with pseudopalisading of tumor cells, leading to diagnose as glioblastoma (WHO grade IV astrocytoma) (Fig. 6a, b). Ki-67 proliferation-related labeling index was very high, at 42.8 %, and most tumor cells showed positive immunostains for both p53 and EGFR (Fig. 6c–e), but a negative staining for isocitrate dehydrogenase 1 (IDH1) (Fig. 6f). Extended focal radiotherapy at a total dose of 50 Gy and one course of chemotherapy comprising carboplatin and etoposide were performed. Nine months after radio-chemotherapy, the patient died of dissemination from the infratentorial tumor through the ventricle space, but residual insular tumor remained unchanged. As permission of autopsy could not be obtained from his family, we could not confirm the histology of these lesions.

The clinical study of the abovementioned case report was approved by the Ethics Committee for Clinical Research of Ehime University Hospital, and informed consent was obtained from the patient prior to initiating the study.

### Discussion

As for the definition of multicentric gliomas, Batzdorf and Malamud described the criteria that multifocal tumors should be distinguished from multicentric tumors [3]. The multifocal tumors result from invasion or growth by established routes such as commissural or tract fibers. Otherwise, such tumors occur by spread via cerebrospinal fluid channels or local metastasis. In contrast, multicentric gliomas occur in a distant site to each other, such as in different lobes or hemispheres, and there is no connecting pathway between these tumors. It is also emphasized that these two tumors occur at different times. Conversely, in a brain autopsy study, Mishra et al. stated that even when tumor connectivity is missing based on macroscopic findings, the possibility of microscopic connectivity cannot be completely excluded [4]. Malcolm et al. suggested that gliomas occurring in both supra- and infratentorial regions may be considered true multicentric gliomas, as they arise the different compartment of the brain without gross or microscopic connections [5]. In the present case, the two tumors arose in different locations of supra- and infratentorial regions (one in the left insula; the other in the cerebellum), and no macroscopic connectivity was apparent on MRI. Although the possibility of local intracerebral metastasis could not be excluded, these two tumors were diagnosed as multicentric gliomas because they showed different histological findings.

Multicentric gliomas more often affect middle-aged men and show rapid tumor progression, and the prognosis is extremely poor. Many reported cases of multicentric gliomas occur in the supratentorial compartment, and occurrence in both supra- and infratentorial regions is very rare [4–7]. To the best of our knowledge, only 11 cases of multicentric gliomas arising above and below the tentorium have been reported, and all exhibited different histological findings [4–12]. Mean patient age is 37.8 years (range, 11–63 years). No clear gender differences have been identified. These tumors arise predominantly in the cerebral hemisphere and cerebellum, and all cerebellar tumors have been located around the fourth ventricle, as in the present case. Histological examination of these 11 patients revealed that both supra- and infratentorial tumors showed high-grade gliomas in five patients. In another five patients, when one tumor located in either supra- or infratentorial region showed high grade, the other located in the different region was low grade (including the present case). In one patient, both tumors displayed low-grade glioma. In the present case, the supratentorial tumor remained low grade, whereas the infratentorial tumor rapidly proliferated within several months. Of the past 11 reports, only one patient has been reported with infratentorial tumor rapidly progressing to a malignant type within a few months after resection of the supratentorial tumor.

The pathogenesis of multicentric gliomas remains unclear. Many hypotheses have been proposed to explain the multicentricity. Willis et al. suggested that multicentric lesions could result from a two-step process [13]. At first, a large area of the brain, or even the entire brain, undergoes neoplastic transformation (initiation), becoming more susceptible to neoplastic growth. In the second stage (promotion), neoplastic proliferation in multiple sites occurs after various stimulations. Genetic and molecular analyses are very useful and important for elucidating the pathogenesis of multicentric gliomas.

Reis et al. investigated genetic changes to compare primary and secondary glioblastomas and reported that these tumors were independent of each other, with showing a different pattern of genetic mutations [14]. Furthermore, the phenotype of these tumors included genetic mutations of tumor-suppressor genes such as p53, phosphatase, and tensin homologue deleted from chromosome 10 (PTEN) and overexpression of EGFR. Watanabe et al. indicated that overexpression of EGFR and mutations of p53 are mutually exclusive, and the alteration of either gene leads to different pathways for the evolution of glioblastoma as the common phenotypic endpoint [15]. Primary glioblastoma thus occurs by a de novo pathway with overexpression of EGFR, whereas secondary glioblastoma develops by a progression pathway in which p53 mutations operate as an initiation step for neoplastic transformations less frequently with EGFR overexpression. In the present case, supratentorial tumor revealed immunoreactivity for p53, whereas EGFR showed a negative reaction. The malignant phenotype derived from this tumor is thus postulated to represent a secondary glioblastoma. However, facts that the infratentorial tumor showed positive immunoreactivity for both p53 and EGFR, displayed wild-type IDH1, and had large ischemic necrosis might make a contradiction if the tumor developed along the progression pathway [15, 16]. In the present case, p53 mutations may have occurred throughout the brain, possibly inducing transformation of the glial cells as described by Willis et al. The supratentorial tumor may have developed from a genetic background of p53 mutations. Taking into consideration the fact that the infratentorial tumor occurred within a short period of less than 4 months, the malignant phenotype may have developed through a de novo pathway. These may be the reasons for positive stainings of both p53 and EGFR in the infratentoral tumor. To elucidate the pathogenesis of multicentric gliomas, further genetic analysis of glioma oncogenesis-related genes including these molecules will be necessary with obtaining more critical information.

## Conclusions

We reported a case of multicentric gliomas occurring in the supra- and infratentorial regions with different histopathology. One tumor was diffuse astrocytoma of the left insula, while the other was glioblastoma of the cerebellum. Immunohistochemical analysis suggested that these tumors might have occurred by different pathways of neoplastic transformation in glial cells.

## Abbreviations

EGFR, epidermal growth factor receptor; Gd, gadolinium; IDH1, isocitrate dehydrogenase 1; MRI, magnetic resonance imaging; PTEN, phosphatase and tensin homologue deleted from chromosome 10.
